# Benefits of biodiverse marine resources to child nutrition in differing developmental contexts in Hispaniola

**DOI:** 10.1371/journal.pone.0197155

**Published:** 2018-05-24

**Authors:** Gheda Temsah, Kiersten Johnson, Thea Evans, Diane K. Adams

**Affiliations:** 1 ICF International, Fairfax, Virginia, United States of America; 2 Bureau for Food Security, United States Agency for International Development, Washington, District of Columbia, United States of America; 3 The DHS Program, Blue Raster, Arlington, Virginia, United States of America; 4 Office of Forestry and Biodiversity, United States Agency for International Development, Washington, District of Columbia, United States of America; Institut de recherche pour le developpement, FRANCE

## Abstract

There is an urgent need for an improved empirical understanding of the relationship among biodiverse marine resources, human health and development outcomes. Coral reefs are often at this intersection for developing nations in the tropics—an ecosystem targeted for biodiversity conservation and one that provides sustenance and livelihoods for many coastal communities. To explore these relationships, we use the comparative development contexts of Haiti and the Dominican Republic on the island of Hispaniola. We combine child nutrition data from the Demographic Health Survey with coastal proximity and coral reef habitat diversity, and condition to empirically test human benefits of marine natural resources in differing development contexts. Our results indicate that coastal children have a reduced likelihood of severe stunting in Haiti but have increased likelihoods of stunting and reduced dietary diversity in the Dominican Republic. These contrasting results are likely due to the differential in developed infrastructure and market access. Our analyses did not demonstrate an association between more diverse and less degraded coral reefs and better childhood nutrition. The results highlight the complexities of modelling interactions between the health of humans and natural systems, and indicate the next steps needed to support integrated development programming.

## Introduction

Coastal ecosystems provide valuable ecosystem services such as protection from storms and beach erosion, habitat grounds for commercially important fish species and other mariculture, and hotspots for marine biodiversity [1] that should contribute positively to human health and nutrition. An estimated 3 billion people worldwide depend on fish as a significant source (20% or more) of animal protein [2]. Fish supply additional essential micronutrients, vitamins, and fatty acids without which an estimated 845 million to 1.39 billion people would be vulnerable to nutrient deficiencies [3].

An estimated 80% of the world’s fish catch that provides these benefits is provided by the 22 to 30 million people employed in small-scale artisanal fisheries [4, 5]. For most of these fishermen, both the income from sales and direct consumption of part of the catch contribute to household health and nutrition. The smaller, low-value fish are more likely to be consumed in the household [5]. These small fish species often have more nutrient value as the whole fish is consumed including bioavailable calcium in the bones and the micronutrient-rich head and viscera [6]. In addition to fish, other nutritive food species, including octopus, sea cucumbers, seaweeds, and bivalves collected along the coast can supplement household diets and/or income to improve health outcomes.

Associations between human health and marine ecosystems has led many to attribute a causal relationship between biodiversity and human welfare (e.g. [7]), but the connection to biodiversity often remains tenuous. For example, improved human health and welfare outcomes are associated with marine protected areas [8–12], but it is not clear if changes in biodiversity were causative. Marine protected areas can increase the biodiversity of fishes and invertebrates within their boundaries and through spill over to adjacent areas [13–15]. However, human health outcomes might instead be due to increases in fish stock biomass (e.g. [16, 17]) independent of an increase in overall biodiversity, or due to interventions associated with the establishment and implementation of the marine protected areas, which often include community education and empowerment (e.g. [18]). However, nutrient composition of fish and other marine food products vary widely such that seafood diversity within a diet should impact nutritional outcomes [3, 6]. Thus, there is a need for improved understanding the importance of marine biodiversity for human health.

In the tropics, coral reefs are particularly diverse and an important source of food and income [19]. An estimated six million small-scale fishers and gleaners use coral reefs globally [20]. Within many small island states, over 50% of the population may fish coral reefs [20]. Millions more rely on coral reefs for tourism-based livelihoods. Yet, coral reefs are being destroyed and degraded worldwide [19, 21] and threatened with increased future losses due to ocean acidification and warming temperatures associated with climate change [22]. Degradation of coral reefs is often accelerated due to increased pollution, dredging, destructive fishing, and sedimentation associated with economic development activities and land-use changes.

As one of society’s most marginalized groups [23, 24], small-scale artisanal fishermen are an often isolated and vulnerable segment of the population that could be left behind as a nation develops its infrastructure, markets and food production and degrades its natural systems [25, 26]. This vulnerability is likely intensified for those who rely on the already threatened and degrading coral reefs. These communities may be poorly positioned to make the transition to replacing ecosystem services with built infrastructure and markets for food, shelter, water purification, fuel, and protection due to lack of personal capital [25]. Many marine resources remain part of a common pool with low to no investment cost for access by an individual, independent of the broader national development context. In contrast, expanded market systems may require more capital or goods from an individual to reap subsequent income and nutritional benefits. Thus, differential development contexts could result in relatively improved health and nutrition outcomes for communities with access to marine resources in poorly developed countries, or relatively poor health and nutrition outcomes for these communities left behind as the rest of the nation develops. Aggregated data that demonstrate large gains in human health through economic development may mask the negative effects of development on these marginalized subpopulations. We will explore these relationships among biodiverse marine resources, human health and nutrition and development in the island of Hispaniola, where coastal communities rely on the surrounding coral reefs in contrasting development contexts.

### Study site: Haiti and the Dominican Republic

#### Socio-economic context

We selected the island of Hispaniola, which consists of Haiti and the Dominican Republic, because of the availability of quality population, health and environmental data in two contrasting national contexts. The large coastline of this landmass increases the probability of geographic proximity between population/health data and marine data. While the population size of the two countries is about the same, their socio-economic characteristics differ vastly. Haiti is the poorest country in the Western Hemisphere and amongst the poorest in the developing world [27]. In 2007, approximately 54 percent of the population in Haiti lived on less than $1 per day [28]. In 2006, only 2.5 percent of the population in the Dominican Republic lived on less than $1 per day [29]. Still, approximately 25 percent of the population in the Dominican Republic lived below the national poverty line in 2006 [29]. For the years considered in this study, the Demographic and Health Surveys (DHS) (Haiti [30]; Dominican Republic [31]) found the following. About 29 percent of children are stunted in Haiti compared to approximately 10 percent in the Dominican Republic. Infant mortality in Haiti is 70 deaths per 1,000 live births compared to 33 in the Dominican Republic. Under-five mortality in Haiti is 102 deaths per 1,000 live births; the commensurate figure for the Dominican Republic is 37 deaths per 1,000 live births. Less than 70 percent of the adult population in Haiti is literate compared to more than 90 percent in the Dominican Republic. Bicycles were the most common mode of transportation in Haiti (18%), with only 5% of households with a car and 3% with a motorcycle, whereas cars (18%) and motorcycles (25%) were the most common modes of transportation in the Dominican Republic.

#### Biophysical environmental context

The state of natural resources in both countries differs considerably–Haiti’s natural terrestrial and marine resources are far more degraded than those of the Dominican Republic. According to the Food and Agriculture Organization [32], about 98 percent of land in Haiti is severely degraded and 90 percent of land in the Dominican Republic is moderately to severely degraded primarily due to deforestation.

Hispaniola is in a priority ecoregion, the Greater Antillean Marine, which includes the coral reef of Haiti and the Dominican Republic, and is considered one of the 43 priority marine ecoregions crucial to the conservation of global biodiversity [33]. However, these reefs are also largely degraded. Coral reefs around Hispaniola consist of near-shore fringing and barrier reefs [34], which make them easily accessible by paddleboat, sailboat or event swimming [35]. Although most of Haiti’s coral structures are intact, they are some of the most overfished reef in the world [19, 35]. Living coral account for less than one-tenth of most surveyed reefs while algae and sponge occupy more than one-half [35]. Haiti is among the nine countries most vulnerable to coral reef degradation, due to high reef dependence, very high threat exposure and very low adaptive capacity [19]. While coral reefs in the Dominican Republic are less degraded than Haiti’s, increases in deforestation, slash and burn agriculture, coupled with changes in irrigation practices and the increase of waste disposal in coastal waters have contributed to the progressive degradation of the country’s reefs [36, 37]. The most direct threats to the coral reefs of the Dominican Republic are intense fishing and beachfront use from tourism [37] as well as soil erosion and sedimentation, road development for tourism, and other industrial development [38].

Coastal resources can play an important role in the nourishment and livelihoods of populations in Hispaniola. In Haiti, coastal resources are the primary source of income and food for an estimated 50,000 fishers and their families [39]; of that nearly all are estimated to rely on fishing coral reefs [20]. Protein from fisheries represents a significant proportion of total animal protein consumption in Haiti (14–15%, in 2007–08 [40]; 7–12%, in 2010 [3]). This may be higher in coastal communities and in times of drought [41]. Since less than 20% (by weight) of Haiti’s fisheries products are oceanic fish, and aquaculture and inland fisheries were negligible in the 2000s [39], it is likely that coastal fish and invertebrates that permanently or transiently rely on reefs compose a large portion of this protein source. Coral reefs are an important source of food and income in some parts on the Dominican Republic as well [41, 42]. Coastal fishing is practiced by about 9,000 fishers in the inland shores; most fishers are concentrated in the poorest regions and fishing is their most important and often only source of livelihood [42]. Again, nearly all of these fishermen fish coral reefs [20]. While fish is an important source of sustenance in Haiti, per capita per day protein intake and caloric consumption of fish and seafood in the country is less than one-half those in the Dominican Republic [43]. So while fishing is more central to many lives in Haiti, the Dominican Republic consumes more fish.

Intense and poorly regulated fishing, in addition to other factors, are threatening fish stocks; the International Union for the Conservation of Nature has classified 21 fish species as threatened in Haiti and 22 in the Dominican Republic [44]. High-value reef-associated commercial species such as spiny lobster, southern red snapper and yellowtail snapper have declined sharply in the Dominican Republic and are well below 1980s levels [42]. Lower-value, smaller reef species have also been heavily fished [35] for subsistence or sold to the urban poor [41]. The Haitian artisanal fisheries are generally not selective, and everything caught, even small bodied damselfish, is used either for human consumption or for bait [45]. Overall, environmental legislation and institutional capacities to enforce protections in Haiti are poor, contributing to unplanned urbanization and the further destruction of natural resources [46]. Coastal areas in the Dominican Republic are protected to some extent; legislation provides some protections for mangroves and regulates fishing and aquaculture [37].

The socioeconomic and biophysical environmental differences described above enable us to explore whether biodiverse marine resources play a vital role in fostering positive child health and nutrition outcomes under differing developmental conditions.

### Pathways between marine biodiversity and health outcomes

**Fig 1:**
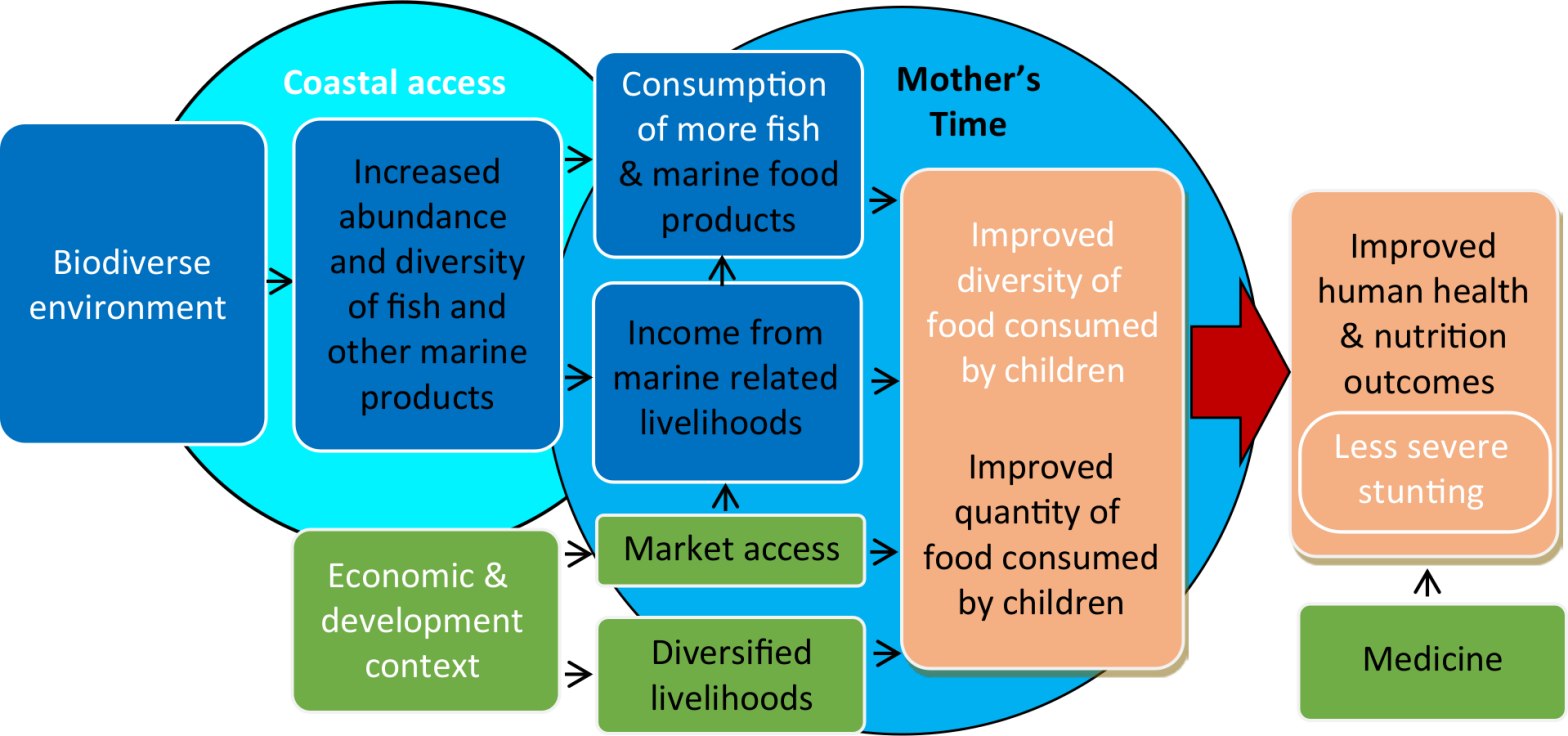
Pathways in which biodiversity and natural resources contribute to human health and nutrition. While we are not able to test all of the connections illustrated due to data limitations, we specifically investigate those in white text. Modified from [49].

In this study, we explore the association between distance to coastline and proxies for marine biodiversity and child health and nutrition outcomes in two countries with different levels of development (Fig 1). Healthy, biodiverse marine environments should provide coastal communities with a diversity and abundance of fish and other marine products for direct consumption and to support livelihoods. Both direct consumption and income affect total caloric intake (food quantity) and dietary diversity (food quality) for children. These are also mediated by access to markets and other livelihood options. Integrated over time, deficits in childhood diets can lead to lasting health outcomes, such as severe stunting. Stunting is multifactorial with other contributing health factors such as access to medicine and incidence of disease, but nutrition can also be significant.

While we are not able to address all of the proposed connections in these pathways (Fig 1), this study does specifically address the following hypotheses. We hypothesize that proximity to the coastline (a determinant of households’ access to marine resources and marine-based livelihoods) opens pathways to better child health and nutrition through diverse diets including fish and shellfish consumption, and subsequent reductions in the incidence of stunting. Because coral reefs are a prominent and biodiverse ecosystem around the island, we also explore the importance of coral reefs, in particular. We hypothesize that more diverse and less degraded coral reefs open pathways to better child health and nutrition. This connection is likely mediated by a greater abundance and diversity of fish and mariculture in diverse, healthy reefs [47], however this explicit connection cannot be tested with available data.

We hypothesize that coastal proximity and marine biodiversity will be less important in shaping child health and nutrition outcomes in more advanced development contexts. Developed road and transportation systems, modern modes of agricultural production and fishing can influence food production, commercialization and consumption in ways that facilitate access to fortified foods, imports, and alternative proteins in addition to influencing household wealth. The consumption of fish and other wild resources may become more influenced by the availability and price of substitutes [48] rather than direct access. Thus, we expect coastal proximity to be a more salient variable in child health and nutrition in Haiti compared to the more developed Dominican Republic.

## Data and methods

We used health and nutritional data from the Haiti DHS 2005–2006 and the Dominican Republic DHS 2007 to evaluate the association between biodiverse marine resources and childhood fish and shellfish consumption, dietary diversity and incidence of severe stunting. Procedures and questionnaires for the DHS surveys have been reviewed and approved by ICF International Institutional Review Board in accordance with the United States Department of Health and Human Service requirements for the “Protection of Human Subjects (45 CFR 46) Any country-specific DHS survey protocols are additionally approved by an ethics review board in the host country. The research complies with the current laws of the country in which they were performed. We did not use more recent datasets to avoid confounding issues caused by the destructive 2010 earthquake and subsequent distribution of aid. DHS surveys are conducted using a two-stage stratified cluster sampling design. The majority of DHS since 2000 are geo-referenced to enable spatial analysis and linking with other geo-referenced datasets. In order to ensure respondents’ confidentiality, all of the GPS coordinates are randomly displaced prior to the public release of the data so that urban clusters are displaced up to 2 kilometers and rural clusters are displaced up to 5 kilometers, with a further 1 percent of rural clusters displaced up to 10 kilometers. To minimize the effect of the displacement of GPS coordinates on our analyses, we include all clusters with a centroid falling within 5km of the coastline which would account for the majority of cases.

A total of 9,998 households participated in the 2005–2006 round of the DHS in Haiti [30]. In the Dominican Republic 32,431 households participated in the 2007 round [31]. The household response rates in Haiti and the Dominican Republic are 99.6 percent and 97 percent, respectively [30, 31].

### Dependent variables

We focus on dietary diversity among children aged 6–35 months and severe stunting among children under the age of five years. We include a measure of fish and shellfish consumption as a dependent variable as well due to the direct linkage to marine resource access. The United Nations Children’s Fund uses various indicators for child nutritional status, such as low birth weight, underweight, wasting, and vitamin A intake [50]. We selected dietary diversity and severe stunting as available DHS indicators of recent child nutritional status and of long-term, integrated nutrition status, respectively, each with impacts on growth and later life outcomes. Dietary diversity reflects the diversity of macro- and micro-nutrient intake [51]. Further, analyses in the Philippines suggest that access to protected marine resources can contribute to an overall improvement in dietary diversity, not just in fish consumption [12]. Unlike wasting and underweight; stunting is an irreversible condition [50], impacting cognitive ability, schooling, as well as health, employment and earnings in adulthood [52–54].

Data on dietary intake, including fish and shellfish consumption, were collected for most recently born children under 3 years of age. Responses are based on a 24 hour recall period. Our definition of dietary diversity is restricted to children between 6–35 months and parallels that of Johnson et al. [49] and Alva et al. [12]. Although the WHO restricts its dietary diversity score to children age 6–23 months [55], we use an expanded age range to explore the associations between marine biodiversity and child nutrition with a larger sample.

Anthropometric data were collected for a subsample of children who are under the age of five. Based on WHO growth standards [55], children who are found to have height-for-age z-scores that are –3 standard deviations (SD) from the median of the reference population are considered to be severely stunted. The reference populations are gender-specific to account for any gender-linked growth patterns. Since stunting is multifactorial and moderate stunting is common, we conservatively analyze severe stunting to reduce the chances of obtaining a spurious result.

To avoid unobserved intra-household correlation in our outcomes of interest, we restrict our study sample to the most recently born child. The sample sizes for the portion of the analyses on dietary diversity are,1,111 children in Haiti and 4,375 children in the Dominican Republic and on stunting are 1,782 for Haiti and 7,204 for the Dominican Republic. The unweighted percent distribution of children across the categorical variables is presented in Table A in S1 File.

### Key explanatory variables

This study explores the association between marine resources and child health and nutrition outcomes using three proxy measures: (1) distance to coast; (2) the diversity of coral reef geomorphology (habitat); and (3) a coral reef threat index near the index child’s community. Distance to coastline provides a measure of access to marine resources in general. For an initial assessment of the role of biodiversity, we use the number of different reef habitats as our proxy of coral reef diversity, as in [56], and use the Reefs at Risk coral reef ‘threat index’ [19] to acknowledge the degradation of reefs. While we would ideally use a direct quantification of total biodiversity and abundance, or that of a harvested taxa, data are not available at the scale of the DHS datasets. Remotely sensed data for geomorphology is available for the entire island to enable pairing with the DHS data. The diversity of reef geomorphology has been shown to correlate with biological habitat richness [57], suggesting that it can be used as a reef-scale biodiversity proxy. While localized reef biodiversity (within a habitat type) is largely determined by microhabitats and non-equilibrium dynamics driven by complex biological interactions and disturbance [58], larger-scale reef biodiversity can be greatly enhanced by geomorphological habitat diversity (e.g. [59]). Fewer species, including rare species, are likely to be shared across multiple habitat types; thus as habitat diversity increases, the cumulative diversity of the reef is also likely to increase (e.g. [56]). Additional geomorphological types increases both habitat and structural diversity since different coral species and structural phenotypes can be found in different geomorphological units, based on light, flow conditions, etc. This is consistent with the habitat heterogeneity-species richness hypothesis (e.g. [60, 61, 62]) in which structurally diverse habitat may provide more niches and thus increase overall species diversity [63, 64]. Thus, while not perfect, geomorphological diversity (hereto also referred to as habitat diversity) is a suitable proxy for species diversity across the scale humans access.

To determine a cluster’s access to the coastline and coral reefs, distances were calculated from the displaced cluster locations to the closest segment of coastline and the closest coral reef. GPS clusters were assigned a coastal tangent location at the closest point on the coastline from the displaced cluster location according to the U.S. Department of State's Office of the Geographer (INR/GGI)—Small Scale International Boundaries (SSIB). The distances are classified into categories in order to reduce the error introduced by the GPS displacement. Of the 332 survey clusters in Haiti, about 50 percent are within 5 km of the coast line in contrast to the Dominican Republic where 25 percent of the 1,425 sampled clusters are within 5 km of the coastline (Table B in S1 File). Each coastal tangent point was buffered at a 5 km radius to account for the potential distance traveled during a fishing journey.

Data on coral reef boundaries and coral reef geomorphology are derived from the Millennium Coral Reef Mapping Project (UNEP-WCMC) [65], the most comprehensive and detailed database of coral reefs for the region. Data on coral reef threat are from the World Resource Institute (WRI) Reefs at Risk Revisited database [19]. Because the significance of coral reef diversity and coral reef degradation for our outcomes of interest is expected to vary with distance to coastline, a categorical measure of distance to coastline is included as a main explanatory variable. Reef geomorphology of proximal reefs and an average local threat index within each buffered 5 km radius were calculated using zonal statistics (Figures A and B in S1 File).

Coral reef geomorphology was classified into the following categories: barrier island, barrier continental, barrier atoll-bank, fringing island, fringing continental, patch island, patch continental, patch atoll-bank, shelf island and shelf continental. From the reef geomorphology maps, we created a habitat diversity index ranging from 0–3 such that zero corresponds to coastline with no coral reef; 1 corresponds to the least diverse reef (1 type); 2 corresponds to moderately diverse reef (2 types) and 3 corresponds to the most diverse reef (3 or 4 types). Landsat 7 images, used to delineate the reef and classify the geomorphology, were collected in 2001–2002. While dredging and other destructive practices can significantly alter the reef geomorphology used here to define habitat types, geomorphology was not likely to significantly change between image acquisition and the DHS surveys (all pre-2010 earthquake).

**Fig 2:**
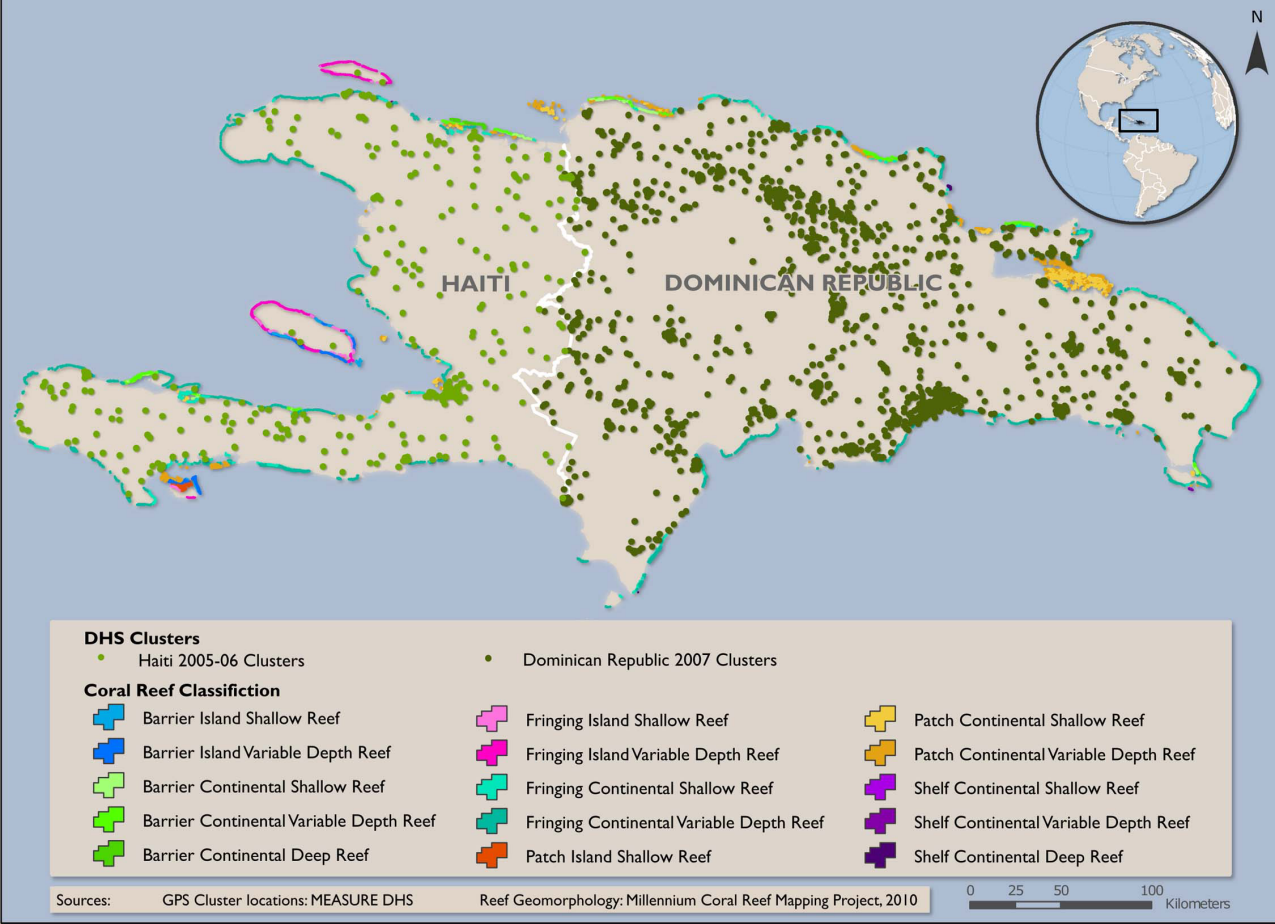
Overlap of DHS samples clusters with coral reef diversity data. Green circles show the locations of clusters in the 2005–2006 Haiti (light green) and 2007 Dominican Republic (dark green) DHS Surveys. Reef geomorphology is indicated by coloring along the coastline, as in the figure legend. Areas without coloring do not have coral reefs. Data from MEASURE DHS and Millennium Coral Reef Mapping Project (UNEP-WCMC) [65].

**Fig 3:**
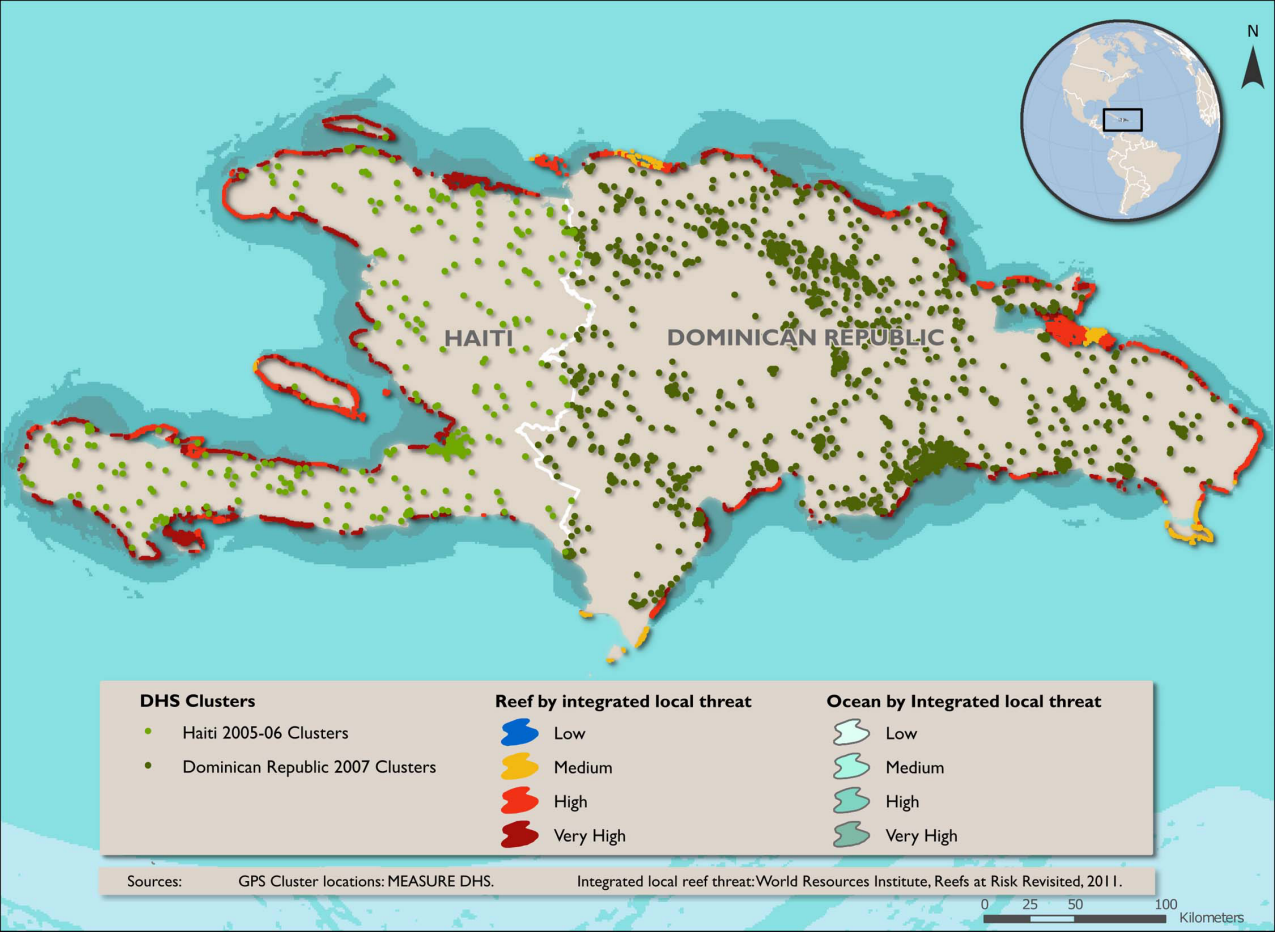
Proximity of DHS sample clusters with coral reef threat data in Hispaniola. Green circles show the locations of clusters in the 2005–2006 Haiti (light green) and 2007 Dominican Republic (dark green) DHS Surveys. The integrated local threat to the reef and oceans are indicated, as specified in the figure legend. Coastline without red to yellow do not have coral reefs. There were no areas of low threat along the coast of Hispaniola. Data from MEASURE DHS and WRI, Reefs at Risk Revisited [19].

We use the World Resources Institute’s coral reef threat indicator as a proxy for the degree of coral reef degradation [19]. The coral reef threat indicator is a measure of local environmental threat to coral reef due to coastal development, marine-based pollution and damage, watershed based pollution, and overfishing/destructive fishing. World Resources Institute classifies reef threat as low, medium, high, and very high. A low threat indicates at or near the potential biodiversity while a high threat would reflect considerably lower biodiversity compared to the potential. Summary statistics of coral reef diversity and coral reef threat within the 5 km buffer of the nearest coastline for the study clusters are presented in Tables C and D in S1 File. About one-quarter of the sample clusters in each country is associated with moderately diverse (2 types) to more diverse reefs (3 or 4 types); however, the majority of cases are designated as high or very high threat (Figs 2 and 3).

### Confounding variables

Childhood health and nutrition is influenced by a number of individual (e.g., gender, age and birth order), household (size and composition), and community-level (e.g. water and sanitation) factors [66, 67]. Because we are interested in the linkages between marine biodiversity and child health and nutrition, we only model confounding variables such as child’s age, mother’s education, household wealth, whether the household owns a mode of transportation, and population density in the household’s location. Maternal education, household wealth and access to a mode of transportation are lower in in Haiti than the Dominican Republic. Although several studies have documented gender differences in child nutrition and food allocation in some contexts [68, 69], we did not detect a statistically significant difference between girls and boys in our outcomes of interest; therefore, to preserve parsimony, sex of the child does not enter our models as a confounding variable.

The extent of growth faltering may vary by age, and the nutrition of children during the first two years is critical for their long-term cognitive and physical development. Additionally, nutritional outcomes such as stunting, which reflect chronic or long-term malnutrition, may be more prevalent among older children. Education and income are likely to determine where households (re)locate. Educated and wealthier families possess both the knowledge and resources to move families closer to locations that have better options for both sustenance and livelihoods. Although households are likely to purchase food in daily and weekly local markets, access to a mode of transport should facilitate access to food not available in the immediate vicinity of where households are located. Urban areas are more likely than rural areas to offer alternative food crops and animal protein, as well as livelihoods other than fishing. Additionally, larger communities are also more likely to offer alternatives to fish consumption. DHS urban and rural classifications are both country and survey specific. To standardize the classification of clusters as urban or rural between the two countries, Landscan 2011 data was used to determine the surrounding population counts and population density. Clusters were buffered by the maximum potential displacement distance (2 km for urban clusters and 10 km for rural clusters) to ensure that the buffered area was certain to contain the true original cluster location. The buffering also accounts for the error in the Landscan data, including the slight shift of the raster due to the applied projection. Clusters were reclassified according to their mean population density.

### Analytical strategy

Because our outcome variables are dichotomous, we use a multivariate logistic regression model to estimate the association between coastal proximity, coral reef diversity and coral reef degradation. We model these associations independently for each country because the DHS surveys were independent and because of the distinct socioeconomic and biophysical country contexts. Thus, we focus on comparisons of the relative benefits of marine resources. The model specification is as follows:

$$\log it[p(Y=1)=\beta_{0}+\beta_{Dis\tan ce}X_{i}+\beta_{coralreef}X_{i}+\sum_{i=1}^{n}\sum_{j=1}^{k}\beta_{j}X_{ij}$$

Where Y is the child health or nutrition outcome variable, *β*_0_ is the intercept; *β*_*Distance*_ is the estimated effect of distance to coastline on the outcome of interest of child i; *β*_*coralreef*_ is the estimated effect of coral reef health on the outcome of interest of child i; and $\overset{n}{\underset{i=1}{\Sigma }}\;\overset{k}{\underset{j=1}{\Sigma }}$represents the confounding variables for k number of confounders and n number of children. Since exact p values are not reported for all models due to space considerations, we indicate p-values as less than 0.1, 0.05 and 0.01.

We include the variables sequentially into the analysis, building progressively complex models. The first model includes distance to coastline, the second incorporates coral reef diversity and the third through sixth models include household level characteristics that are likely to have a confounding effect (mother’s education, household wealth, population density, and household ownership of a mode of transportation). We do not include coral reef diversity and coral reef threat in the same model because of overlapping categories between the two (coastline with no coral reef). We are unable to include an interaction of coral reef diversity and threat because this would result in a loss of statistical power due to few cases (refer to Tables C and D in S1 File). Pooling the data from both countries enhances sample size; however this masks country differences (results not shown here but available upon request). Since coastal communities and poorer households may be particularly dependent on marine resources, we re-run the analyses restricting the sample to coastal households and conduct another analysis with a sample restricted to poor households in order to maximize the potential impact of marine biodiversity variables on child health and nutrition outcomes. The latter two analyses control for the same confounders as previous models. All analyses are conducted using Stata version 12 and results are presented as odds ratios. All models include sampling weights.

## Results

Table 1 illustrates descriptive statistics on severe stunting and dietary diversity by coastal proximity, coral reef habitat diversity, coral reef threat and confounding factors. In Haiti, children who live less than 5 km from coastline are the least likely to be severely stunted compared to those living further from the coast (Pearson chi-squared test, p = 0.024). The percentage of severely stunted children increases with distance from coast (Cochran-Armitage trend test, p = 0.005). Dominican Republic had the highest percentage of severe stunting in the groups closest to the coast and those furthest from the coast, with the lowest 5–10 km from the coastline, but these were not significantly different (Pearson chi-squared test, p = 0.195; Table 1; Panel A of Figure C in S1 File).

**Table 1:** Percent of severe stunting and dietary diversity in Hispaniola by key explanatory variables.

Variables	Categories	Haiti DHS 2005–2006		Dominican Republic DHS 2007	
		Severe Stunting	Dietary diversity	Severe Stunting	Dietary diversity
Distance to coastline					
	Less than 5 km	3.88	38.48	1.76	73.59
	5–10 km	6.79	27.29	0.67	84.64
	11–20 km	7.14	35.37	1.61	71.00
	More than 20 km	7.53	46.09	1.46	75.71
Coral reef habitat diversity					
	No coral reef	5.30	39.53	1.30	75.79
	One type	6.10	41.18	1.47	76.83
	Two types	4.75	28.02	1.60	74.52
	Three or four types	15.40	39.36	1.00	73.54
Coral reef threat					
	No coral reef	5.55	39.68	1.33	75.75
	Low	…	…	…	…
	Moderate	…	…	0.89	73.54
	High	8.20	37.83	1.81	76.54
	Very high	5.53	37.42	1.40	76.31
Child’s age in months					
	< 6 months	3.2	…	0.2	…
	6–8	6.0	22.0	0.3	45.8
	9–11	4.8	42.7	1.4	72.3
	12–17	6.0	45.1	1.9	77.7
	18–23	8.3	46.6	3.4	81.5
	24–35	5.9	30.8	1.3	83.7
	36–47	7.9	…	1.1	…
	48–59	3.3	…	1.1	…
Mother's educational attainment					
	No education	8.82	32.71	2.04	61.89
	Primary	5.70	39.34	1.70	73.91
	Secondary or higher	2.56	42.05	1.18	78.36
Household wealth index					
	Poorest	10.47	34.26	2.58	72.05
	Poorer	6.14	39.06	1.51	72.05
	Middle	5.66	43.30	1.15	80.83
	Richer	3.84	33.28	1.08	75.79
	Richest	2.38	42.17	0.27	83.33
Population density quintiles					
	Lowest population density	12.53	46.41	1.89	72.56
	2nd quintile	4.49	46.68	1.48	73.00
	3rd quintile	6.03	32.88	1.07	72.31
	4th quintile	6.08	41.30	1.60	74.77
	Most densely populated	2.95	31.04	1.34	80.04
Household owns a mode of transportation					
	Does not own a mode of transportation	6.47	36.21	1.37	75.10
	Owns a mode of transportation	3.17	44.34	1.46	77.46
Total		5.77	38.02	1.42	76.14

Notes: Weights included. Sample is restricted to most recently born children to avoid intra-household correlations in the selected outcome.

The association between coastal proximity and dietary diversity is unclear. In Haiti, children who live within 5–10 km of the coastline have the lowest levels of dietary diversity (Pearson chi-squared test, p = 0.002), in contrast to the Dominican Republic where the comparable group of children have the highest levels of dietary diversity (Pearson chi-squared test, p < 0.001; Table 1; Panel B of Figure C in S1 File). However, dietary diversity does not change consistently with distance from coastline for either country (Cochran-Armitage trend test, p = 0.127 Haiti, p = 0.920 Dominican Republic).

In both countries, dietary diversity and severe stunting do not change consistently with increasing reef habitat diversity (Cochran-Armitage trend test, Haiti: p = 0.274 stunting, p = 0.059 dietary diversity; Dominican Republic: p = 0.902 stunting, p = 0.994 dietary diversity) nor with reef threat (Cochran-Armitage trend test, Haiti: p = 0.362 stunting, p = 0.499 dietary diversity; Dominican Republic: p = 0.753 stunting, p = 0.663 dietary diversity; Table 1; Figures D and E in S1 File). Stunting levels in Haiti are highest among children who live in proximity of the most diverse coral reef habitat (Pearson chi-squared test, p = 0.046). There appears to be little to no difference between stunting levels by reef habitat diversity in the Dominican Republic (Pearson chi-squared test, p = 0.771; Panel A of Figure D in S1 File). Some of these differences could be due to or masked by confounding factors. We explore these associations in multivariate analyses which control for confounding variables described above.

### Dietary diversity

The association between distance to coastline and dietary diversity is in the expected direction in Haiti (Table 2) but reversed in the Dominican Republic (Table 3) in the multivariate analyses. In Haiti, children living 5–10 km from the coastline are 40 percent less likely to have a diverse diet (p<0.05) compared to children within 5 km of the coast (Model 1 in Table 2). However, controlling for confounding variables washes away the statistical significance of this association (Model 7 in Table 2). In the Dominican Republic, children living 5–10 km from the coast are about 2.4 times as likely to have a diverse diet compared to children living within 5 km of the coast (p<0.01), net of the effects of reef habitat diversity and confounding factors (Model 7 in Table 3). Additionally, children living more than 20 km from the coast are about 1.4 times more likely to have a diverse diet (p<0.05).

**Table 2:** Estimated odds ratios for the logistic regression model of the relationship between habitat diversity and dietary diversity among children age 6–35 months, Haiti DHS 2006.

Variables	Model 1	Model 2	Model 3	Model 4	Model 5	Model 6	Model 7
**Distance to coast line (ref.: < 5 km)**							
5–10 km	0.605**	0.598**	0.597**	0.634*	0.641*	0.679	0.681
11–20 km	0.872	0.772	0.745	0.814	0.805	0.676*	0.676*
More than 20 km	1.401*	1.224	1.172	1.304	1.294	0.914	0.911
**Index of habitat diversity (ref.: one type of reef)**							
No coral reef		0.861	0.886	0.892	0.884	0.876	0.873
Two types of reef		0.582**	0.579**	0.566***	0.580**	0.615**	0.616**
Three or more types of reef		0.865	0.87	0.989	0.974	0.629	0.624
**Constant**	2.786***	2.966***	0.734*	0.308***	0.309***	0.277***	0.274***
**Wald Chi2 (df)**	17.11 (3)	18.9 (6)	175.6 (10)	187.5 (12)	206 (16)	219.1 (20)	218.5 (21)
**N**	4,375	4,375	4,375	4,375	4,375	4,372	4,353

Notes: Weights included. Models 1–2 have no controls; Model 3 controls for child’s age’ Model 4 controls for mother’s education; Model 5 controls for household wealth; Model 6 controls for population density; and Model 7 controls for mode of transportation.

*** p<0.01

** p<0.05

* p<0.1

**Table 3:** Estimated odds ratios for the logistic regression model of the relationship between habitat diversity and dietary diversity among children age 6–35 months, Dominican Republic DHS 2007.

Variables	Model 1	Model 2	Model 3	Model 4	Model 5	Model 6	Model 7
**Distance to coast line (ref.: < 5 km)**							
5–10 km	1.978***	2.020***	2.235***	2.241***	2.258***	2.392***	2.392***
11–20 km	0.879	0.87	0.828	0.889	0.922	1.016	1.02
More than 20 km	1.118	1.12	1.152	1.226	1.242*	1.395**	1.400**
Index of habitat diversity (ref.: one type of reef)							
No coral reef		0.862	0.898	0.907	0.885	0.863	0.866
Two types of reef		0.886	0.867	0.836	0.846	0.875	0.86
Three or more types of reef		0.84	0.856	0.869	0.888	0.99	0.977
Constant	0.621***	0.760**	0.365***	0.271***	0.257***	0.389**	0.390**
Wald Chi2 (df)	10.33 (3)	15.32 (6)	37.8 (10)	40.17 (12)	43.72 (16)	6 1(20)	61.45 (21)
N	4,375	4,375	4,375	4,375	4,375	4,372	4,353

Notes: Weights included. Models 1–2 have no controls; Model 3 controls for child’s age’ Model 4 controls for mother’s education; Model 5 controls for household wealth; Model 6 controls for population density; and Model 7 controls for mode of transportation.

*** p<0.01

** p<0.05

* p<0.1

The importance of coral reef habitat diversity differs by country context and is not in the expected direction. Net of distance to coastline and other confounding factors, Haitian children living in communities in which coral reefs are moderately diverse (2 types) are about 40 percent *less* likely to have a diverse diet than children living in communities in which coral reef are the least diverse (p<0.05) (Model 7 in Table 2). The chances that a child consumes a diverse diet in the Dominican Republic do not seem to be associated with coral reef habitat diversity (Table 3).

In both countries, coral reef threat is not a statistically significant predictor of child dietary diversity (Tables E and F in S1 File). Pooling the data from both countries to address the possible effect of lack of variance did not change the results.

### Fish and shellfish consumption

We explore associations with fish and shellfish consumption (hereto referred to as fish consumption), as it is a dietary factor that might connect access to coastal resources and coral reefs with human health and nutrition. In Haiti, children at all distances greater than 5 km from the coastline were over 50% less likely to have consumed fish (p < 0.05) (Table 4), whereas in the Dominican Republic, only those children greater than 20 km from the coastline had a significantly reduced likelihood of consuming fish (~30% reduction, p < 0.05), net of all cofounding variables (Table 5). The likelihood of fish consumption was not significantly associated with habitat diversity.

**Table 4:** Estimated odds ratios for the logistic regression model of the relationship between fish consumption, coastal proximity and habitat diversity among children age 6–59 months, Haiti DHS 2005–2006.

Variables	Model 1	Model 2	Model 3	Model 4	Model 5	Model 6	Model 7
**Distance to coast line (ref.: < 5 km)**							
5–10 km	0.392**	0.413**	0.441**	0.435**	0.445**	0.471*	0.483*
11–20 km	0.424***	0.415***	0.420***	0.409***	0.352***	0.314***	0.318***
More than 20 km	0.469***	0.433***	0.429***	0.419***	0.376***	0.302***	0.295***
**Index of habitat diversity (ref.: one type of reef)**							
No coral reef		1.536	1.644*	1.623*	1.466	1.457	1.435
Two types of reef		0.979	1.025	1.048	1.143	1.247	1.282
Three or more types of reef		1.447	1.565	1.546	1.287	1.042	1.004
**Constant**	0.266***	0.243***	0.0493***	0.0466***	0.0591***	0.0688***	0.0690***
**Wald Chi2 (df)**	15.61 (3)	16.74 (6)	30.84 (10)	32.46 (12)	44.63 (16)	55.99 (20)	61.84 (21)
**N**	1,145	1,145	1,145	1,145	1,145	1,145	1,145

Notes: Weights included. Models 1–2 have no controls; Model 3 controls for child’s age’ Model 4 controls for mother’s education; Model 5 controls for household wealth; Model 6 controls for population density; and Model 7 controls for mode of transportation.

*** p<0.01

** p<0.05

* p<0.1

**Table 5:** Estimated odds ratios for the logistic regression model of the relationship between fish consumption and habitat diversity among children age 6–59 months, Dominican Republic DHS 2007.

Variables	Model 1	Model 2	Model 3	Model 4	Model 5	Model 6	Model 7
**Distance to coast line (ref.: < 5 km)**							
5–10 km	1.035	1.04	1.059	1.061	1.053	1.046	1.038
11–20 km	1.341	1.343	1.35	1.335	1.313	1.195	1.19
More than 20 km	0.824	0.8	0.808	0.804	0.794	0.726**	0.724**
**Index of habitat diversity (ref.: one type of reef)**							
No coral reef		1	1.003	1.007	1	1.023	1.025
Two types of reef		1.137	1.126	1.125	1.117	1.053	1.051
Three or more types of reef		1.133	1.134	1.13	1.123	1.01	1.005
**Constant**	0.212***	0.211***	0.130***	0.119***	0.120***	0.145***	0.146***
**Wald Chi2 (df)**	7.05 (3)	8.914 (6)	18.7 (10)	19.78 (12)	22.12 (16)	29.77 (20)	30.6 (21)
**N**	4,259	4,259	4,256	4,256	4,256	4,254	4,237

Notes: Weights included. Models 1–2 have no controls; Model 3 controls for child’s age’ Model 4 controls for mother’s education; Model 5 controls for household wealth; Model 6 controls for population density; and Model 7 controls for mode of transportation.

*** p<0.01

** p<0.05

* p<0.1

### Severe stunting

Tables 6 and 7 display the regression results for our models of coastal proximity, coral reef habitat diversity and severe stunting in Haiti and the Dominican Republic, respectively. In Haiti, children who live farther from the coastline are more likely to be severely stunted than children who live within 5 km (Model 1 in Table 6). This persists until wealth is included in the model (Model 5 in Table 6), suggesting an interaction between distance to coast and wealth. Net of reef habitat diversity and confounding variables, children who live 5–10 km away are about twice as likely to be severely stunted than children who live within 5 km (p<0.1).

The results for coral reef habitat diversity run counter to our expectations that more diverse coral reef are associated with better child health outcomes. Haitian children near very diverse (3 or 4 types) coral reef habitat are about 4.6 times more likely to be severely stunted than children living in communities in which reef habitat is the least diverse (1 type of reef) (p<0.01, Table 6). However, this association loses its significance when we control for population density and mode of transportation (Models 6 and 7 in Table 6).

**Table 6:** Estimated odds ratios for the logistic regression model of the relationship between coral reef habitat diversity and severe stunting among children age less than 5 years, Haiti DHS 2005–2006.

Variables	Model 1	Model 2	Model 3	Model 4	Model 5	Model 6	Model 7
**Distance to coast line (ref.: < 5 km)**							
5–10 km	1.804	1.991*	2.005*	1.872	1.843	2.157*	2.143*
11–20 km	1.905**	2.124**	2.210**	1.851**	1.524	1.544	1.543
More than 20 km	2.017**	2.333***	2.359***	1.953**	1.644	1.487	1.503
**Index of habitat diversity (ref.: one type of reef)**							
No coral reef		0.809	0.81	0.821	0.803	0.743	0.751
Two types of reef		0.985	1.049	1.203	1.273	1.254	1.239
Three or more types of reef		4.614***	4.658***	3.854**	3.101*	1.961	2.052
**Constant**	0.0404***	0.0375***	0.0189***	0.0305***	0.0437***	0.0700***	0.0683***
**Wald Chi2 (df)**	6.753 (3)	14.03 (6)	23.49 (13)	31.92 (15)	38.51 (19)	54.1 (23)	58.31 (24)
**N**	1,782	1,782	1,782	1,782	1,782	1,782	1,781

Notes: Weights included. Models 1–2 have no controls; Model 3 controls for child’s age’ Model 4 controls for mother’s education; Model 5 controls for household wealth; Model 6 controls for population density; and Model 7 controls for mode of transportation.

*** p<0.01

** p<0.05

* p<0.1.

**Table 7:** Estimated odds ratios for the logistic regression model of the relationship between coral reef habitat diversity and severe stunting among children age less than 5 years, Dominican Republic DHS 2007.

Variables	Model 1	Model 2	Model 3	Model 4	Model 5	Model 6	Model 7
**Distance to coast line (ref.: < 5 km)**							
5–10 km	0.375*	0.377*	0.379*	0.388	0.397	0.41	0.364
11–20 km	0.912	0.913	0.934	0.881	0.723	0.796	0.78
More than 20 km	0.827	0.829	0.841	0.812	0.722	0.805	0.794
**Index of habitat diversity (ref.: one type of reef)**							
No coral reef		0.965	1.009	1.001	0.958	0.961	0.994
Two types of reef		1.068	1.128	1.157	1.075	1.2	1.118
Three or more types of reef		0.671	0.676	0.663	0.584	0.685	0.652
**Constant**	0.0179***	0.0182***	0.0025***	0.0036***	0.0044***	0.0039***	0.0037***
**Wald Chi2 (df)**	3.006 (3)	3.771 (6)	30.57 (13)	46.98 (15)	67.89 (19)	68.15 (23)	76.24 24)
**N**	7,204	7,204	7,204	7,204	7,204	7,199	7,171

Notes: Weights included. Models 1–2 have no controls; Model 3 controls for child’s age’ Model 4 controls for mother’s education; Model 5 controls for household wealth; Model 6 controls for population density; and Model 7 controls for mode of transportation.

*** p<0.01

** p<0.05

* p<0.1.

In contrast to Haiti, the association between distance to coastline and the likelihood of stunting is negative in the Dominican Republic. Net of reef habitat diversity and confounding variables (Model 3 in Table 7), children living between 5–10 km of the coast are about 63 percent less likely to be severely stunted (p<0.1). But this association loses significance when the models control for confounding factors other than the child’s age. Coral reef habitat diversity is not a statistically significant correlate of severe stunting in the Dominican Republic.

In both countries, coral reef threat is not a statistically significant correlate of child stunting (Tables G and H in S1 File). Because the lack of variation in coral reef threat may partially explain this non-finding, we pooled the data from both countries; however, this did not change the results.

### Coastal and poorer households

Coastal communities and poorer households may be more dependent on marine resources for sustenance and livelihoods. We re-run two additional sets of severe stunting and dietary diversity analyses that are restricted to households living within 5 km of the coastline or to households in the lowest and second lowest wealth quintiles, controlling for distance to coastline and confounding factors. Although odds ratios changed slightly, the patterns and relationships describe above remained the same for all re-analyses with the restricted groups (Tables I–N in S1 File). The only exception is the association between habitat diversity and severe stunting in Haiti. Restricting the sample to households living closest to the coastline, the most diverse coral reefs (3 or 4 types) are associated with odds of severe stunting that are about 5 times that of children living in communities in which coral reef are the least diverse, net of all confounding variables (p<0.05) (Model 5 of Table K in S1 File).

## Discussion

### Development context

Despite the intuitive understanding of the importance of natural resources and biodiversity for human health, empirical evidence supporting these contentions is limited [25]. We analyze the association between tropical marine resources and child health and nutrition in Haiti and the Dominican Republic–two countries with strikingly different development contexts. We hypothesize that coastal proximity and intact marine ecosystems are likely to be important in opening up pathways to better child health and nutrition outcomes. However, we expect these relationships to be mediated by the development context.

Our analyses support our hypothesis that coastal proximity and marine biodiversity will be less important in shaping child health and nutrition outcomes in a more advanced development context. Our findings illustrate that in Haiti, where terrestrial resources are severely degraded, agriculture is not a profitable sector, and road systems are underdeveloped, proximity to the coastline is important for child health and nutritional outcomes. Children within 5 km of the coastline had the lowest likelihood of being severely stunted and highest likelihood of having consumed fish. On the other hand, in the Dominican Republic, where terrestrial resources support livelihoods other than fishing, provide affordable substitutes for fish, and more developed road systems facilitate access to markets, coastal proximity is less important. Coastal children did not have any advantages with regard to stunting, dietary diversity and fish consumption.

We suggest that the relative advantages or disadvantages for coastal children are reflective of the broader development context rather than household poverty context. Associations with dietary diversity and fish consumption were robust to (i.e. not altered by) inclusion of household wealth (Model 4 in Tables 2–4). Associations with severe stunting were sensitive to wealth, especially in Haiti, suggestive of an interaction (Model 5 in Tables 6 and 7). However, Haitian children living within 5 km of the coast still had the lowest likelihood of severe stunting after the inclusion of wealth, but it was no longer significant (Model 5 in Table 6).

Our argument that coastal proximity is particularly important in contexts lacking substitutes for marine resources may be strengthened by incorporating a control for land cover. The interior of Haiti is bare in contrast to the lusher interior of the Dominican Republic (Figure F in S1 File). The agriculturally rich areas of Haiti are mainly in the coastal plains; the mountainous areas are deforested and suffer from soil erosion. Proximity to fertile plains and marine resources could also explain better dietary diversity among children living in coastal areas in Haiti.

Another interesting pattern of childhood nutrition emerges with distance from the coastline, net of all confounding factors. Children 5–10 km from the coast often have the worst odds of a favorable health or nutrition outcome in Haiti and the best in the Dominican Republic (Tables 2–7). We propose a conceptual model in which differential ranges in market access in the two countries can account for the dramatic relative changes in these ‘in-between’ communities that are not likely to have direct access to either marine or inland/forest resources. Where food products remain primarily with the local communities, the potential for access to both marine and agriculture products is low and those with immediate access have the best health and nutrition outcomes. Where products are more readily traded over larger distances, the ‘in-between’ communities may have the best access to both products and thus have the best health and nutrition outcomes. In support of the hypothesis, trends in the likelihood of fish and shellfish consumption (a more direct measure of marine resource use) reflect more localized, coastal only consumption in Haiti with a dramatic drop off in consumption greater than 5 km from the coast. There is a more even pattern of fish consumption in the Dominican Republic, with equal likelihoods of consumption in all but the furthest distance category (Tables 4 and 5). Thus, it appears that while all children have improved health and nutrition outcomes in the Dominican Republic, coastal communities have not benefited to the same extent as communities further inland.

It is possible that as the Dominican Republic continues to develop, coastal communities will once again have equal or better outcomes than inland communities. In other more developed Caribbean countries, such as the Bahamas, Barbados, Montserrat, and Trinidad and Tobago, decreased reliance on extractive fishing as the sole source of household income has reduced and nearly eliminated the population living with unmet basic needs, such as food, utilities and employment [70]. Coastal communities in Jamaica, Belize and Guyana may be more comparable to that described for the Dominican Republic, as the Caribbean Regional Fisheries Mechanism [70] found that 40–50% of fishers’ households were considered vulnerable or poor, with multiple unmet basic needs. Many of these poor households relied on fisheries for all or most of their income.

### Associations with biodiversity

The results demonstrating an association between coral reef habitat diversity and our health and nutrition outcomes are counter to our expectations; more diverse coral reef habitats are associated with poorer child health and nutrition outcomes in Haiti (Table 6; Table K in S1 File). However, this counter-intuitive correlation appears to be driven by low sample size (7 clusters, Table C in S1 File) in the most diverse reef category and one cluster with a particularly high level of severe stunting. By omitting the children in this cluster from the analysis, the association between the most diverse coral reef and stunting is negative (odds ratio of 0.73) and statistically non-significant. This reversal can be partially explained by the characteristics of the omitted cluster: compared to other clusters within 5 km of the coast, the coral reefs within its proximity are highly degraded, 60 percent of its mothers are illiterate, 80 percent of its households rank in the poorest two wealth quintiles, and the cluster is in the lowest population density quintile indicating that it is rural. This area may be an area of opportunity for integrated conservation and health programming.

A similar geographic restriction cannot explain the dietary diversity results, where Haitian children living near two types of reef were significantly less likely to have a diverse diet (Table 2; Table I in S1 File). However, our proxy measure of habitat diversity does not take into account remaining or actual biodiversity. Haiti’s coral reef structures are intact but they are largely overfished, suggesting the marine equivalent of the “empty forest syndrome” [71]. While most studies underscore a positive association between habitat heterogeneity and species diversity, heavily impacted systems may not follow this association. Species richness and abundance may be determined by the percentage of coral reef cover [47] for which we do not control, and for which we do not have sufficient data.

We attempted to address degradation with the Reefs at Risk coral reef threat, however, sample size restrictions limited our ability to interact reef diversity with the extent to which they are degraded. The lack of statistical significance of habitat diversity in the Dominican Republic, and coral reef threat in both countries could be driven by a lack of statistical power or very little variance; most of the coral reefs are highly to very highly degraded in both contexts.

These limitations suggest that quantitative biodiversity data are needed at national scales to address this problem appropriately; alternatively, it may be that marine biodiversity is not integral to child health and nutrition, even in coastal and poor communities. Other factors, both on the household and community levels, may be better predictors of these development outcomes. A study of environmental and social correlates of child hunger found that household factors account for more of the variation in child hunger than environmental variables, which the authors attribute to the greater potential for measurement error for environmental variables [72]. Environmental variables may be still important, but diversity may not be the right one. Available fish biomass may better predict childhood dietary outcomes. We do not consider fish biomass here as there are not datasets with the appropriate resolution and fish biomass may vary independently of biodiversity–our variable of interest.

### Modeling and data limitations

The results of our analyses illustrate the challenges of modelling complex associations between the health of natural systems and that of human systems. There are often many intervening factors and feedback effects which our models do not capture, partially due to data limitations.

Some specific limitations should be highlighted. First, the cross-sectional nature of our data limits our ability to draw conclusions as to causal pathways. We argue that marine biodiversity impacts health and nutrition directly in terms of consumption of marine foods, and indirectly in terms of income generated from fishing, gleaning and mariculture. We modeled one of these pathways and find strong and statistically significant associations between coastal proximity and fish consumption in Haiti (Table 4): children living in communities more than 5 km from the coastline are less likely to consume fish. In the Dominican Republic the likelihood of consuming fish is largely unrelated to coastal proximity (Table 5). However, we could not empirically demonstrate the correlation between fish consumption and child health; fish consumption is associated with lower odds of severe stunting in both countries but the associations are not significant. Additional data on what percentage of locally caught fish are consumed versus sold and supplementation with imported fish would enhance our understanding of whether and how marine resources are associated with child health and nutrition.

Second, our measure of coastal proximity does not take into account the nature of the shoreline, which will determine the extent to which households can access marine resources, including coral reefs. Populations living on sandy beaches have direct access to marine resources, in contrast to those living atop a high altitude rocky cliff, who despite being at the same distance from the coast, will not have the same access.

Third, the displacement of the DHS clusters impacts proximity to the coastline, and potentially the effect of our proxies of marine biodiversity since they are only meaningful when considered in conjunction with coastal proximity. We attempted to address this issue by adopting a threshold distance of 5 km and a categorical measure of proximity. The 5 km threshold should be adequate since most clusters, with the exception of 1 percent of rural clusters, are displaced between 2 and 5 km. However, it is conceivable that some clusters may fall outside of the 5 km threshold.

## Conclusion

The combination of remotely sensed data with household-level DHS surveys has provided an island-wide picture of the relative benefits of marine resources in differing development contexts. While coastal communities had better childhood outcomes than some inland communities in Haiti, coastal proximity was not associated with improved outcomes in the Dominican Republic. We propose an additive conceptual model in which market distribution distance from the coastline determines the trends in child nutrition. Patterns of fish consumption support this model. The observed transition in the effects of proximity to natural resources with development partially supports Myers and colleagues [25] model in which the [rural] poor lag behind. However, even though coastal Dominican children lagged behind their inland counterparts, they still had improved outcomes compared to Haitian children. If other Caribbean countries serve as indicators, as development continues, fishing communities may continue to be buoyed up and escape poor and vulnerable conditions [70]. The role of biodiversity in mediating these trends remains unclear. Datasets of marine biodiversity at the scale of the DHS surveys will be needed to truly address this question. Currently, our insights are limited by incomplete and coarse data or the use of proxies.

The research performed complies with the current laws of the country in which they were performed.

## Supporting information

S1 File Supporting information. (DOCX)
