# Supplementary material for: Benefits of biodiverse marine resources to child nutrition in differing developmental contexts in Hispaniola
Source: PLoS One. 2018 May 24;13(5):e0197155. doi: 10.1371/journal.pone.0197155 (PMC5967791; doi:10.1371/journal.pone.0197155)
Supplement: S1 File — (DOCX) [file pone.0197155.s001.docx]

**Supplementary Information**

**MARINE BIODIVERSITY AND CHILD NUTRITION IN THE ISLAND OF HISPANIOLA**

# **Figure A** Illustration of calculation of distance to coastline and coral reef habitat diversity


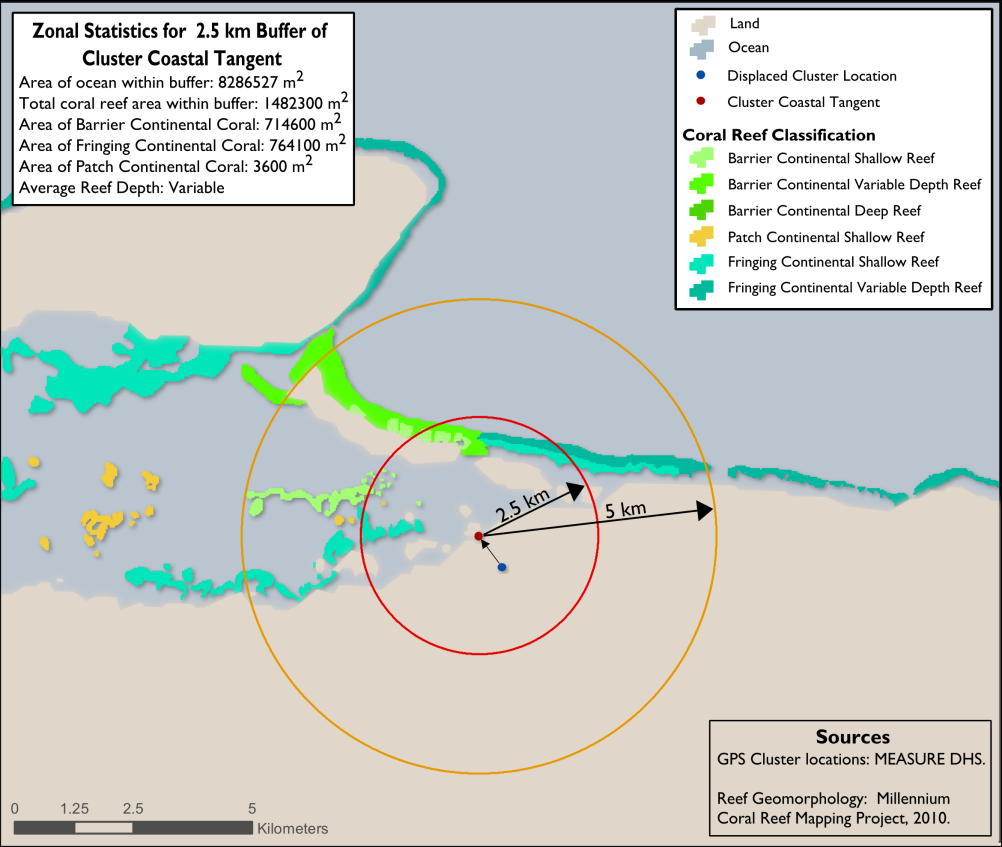


# **Figure B** Illustration of calculation of distance to coastline and coral reef threat


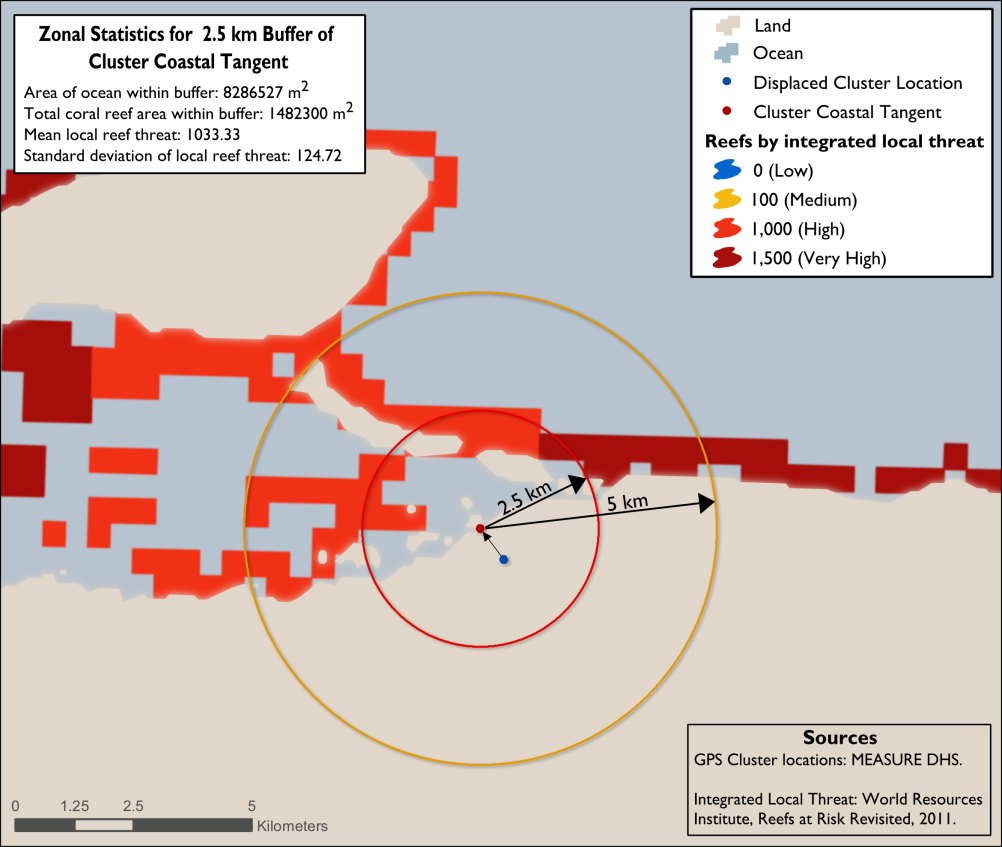


**Figure C**

Panel A. Severe stunting by coastal proximity, Haiti and the Dominican Republic

0.00

1.00

2.00

3.00

4.00

5.00

6.00

7.00

8.00

Haiti DHS 2005-2006

DR DHS 2007

Less than 5 km

5-10 km

11-20 km

More than 20 km

*Source*: DHS

# Panel B. Dietary diversity by coastal proximity, Haiti and the Dominican Republic

0.00

10.00

20.00

30.00

40.00

50.00

60.00

70.00

80.00

90.00

Haiti DHS 2005-2006

DR DHS 2007

Less than 5 km

5-10 km

11-20 km

More than 20 km

# *Source*: DHS

**Figure D**

Panel A. Severe stunting by coral reef diversity, Haiti and the Dominican Republic

0.00

2.00

4.00

6.00

8.00

10.00

12.00

14.00

16.00

18.00

Haiti DHS 2005-2006

DR DHS 2007

No coral reef along

nearest coastline

One type

Two types

Three or four types

*Source*: DHS; UNEP Millenium Coral Reef Mapping Project

# Panel B**.** Dietary diversity by coral reef diversity, Haiti and the Dominican Republic

0.00

10.00

20.00

30.00

40.00

50.00

60.00

70.00

80.00

90.00

Haiti DHS 2005-2006

DR DHS 2007

No coral reef along

nearest coastline

One type

Two types

Three or four types

# *Source*: DHS; UNEP Millenium Coral Reef Mapping Project

**Figure E**

Panel A. Severe stunting by coral reef threat, Haiti and the Dominican Republic

0.00

1.00

2.00

3.00

4.00

5.00

6.00

7.00

8.00

9.00

Haiti DHS 2005-2006

DR DHS 2007

No coral reef along

nearest coastline

Moderate

High

Very high

*Source*: DHS; WRI Reefs at Risk Indices 2010

# Panel B. Dietary diversity by coral reef threat, Haiti and the Dominican Republic

*Source*

2010

DHS; WRI Reefs at Risk Indices

:

0.00

10.00

20.00

30.00

40.00

50.00

60.00

70.00

80.00

90.00

Haiti DHS 2005-2006

DR DHS 2007

No coral reef along

nearest coastline

Moderate

High

Very high

# **Figure F** Land cover classification in the Island of Hispaniola


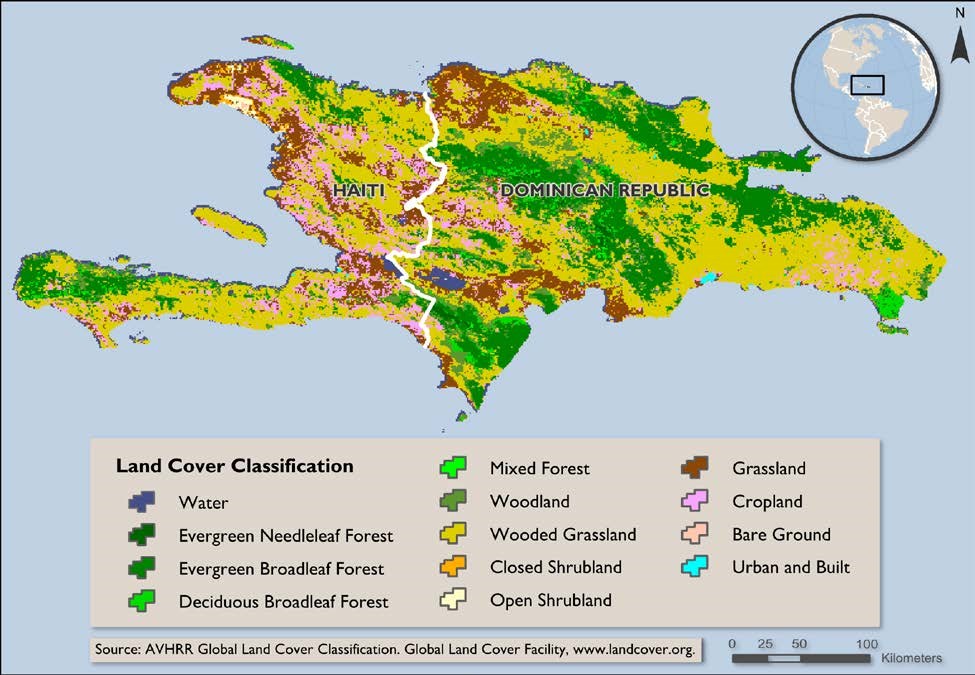


**Table A.** Unweighted percentage distribution of analytical sample by categorical variables used in analysis^(1)^

Haiti Dominican Republic

DHS 2005-2006 DHS 2007

|  | < 5 years | 6-35 months | < 5 years | 6-35 months |
| --- | --- | --- | --- | --- |
| Variables |  |  |  |  |
| Distance to coastline  Less than 5 km | 42.65 | 42.68 | 23.74 | 23.86 |
| 5-10 km | 12.85 | 12.72 | 11.88 | 12.14 |
| 11-20 km | 23.57 | 22.82 | 11.70 | 11.73 |
| More than 20 km | 20.93 | 21.78 | 52.68 | 52.27 |
| Coral reef diversity index (5 km  coastal buffer)  No coral reef in nearest coastline | 22.73 | 23.08 | 26.78 | 27.47 |
| One type | 56.06 | 56.53 | 50.25 | 50.15 |
| Two types | 18.57 | 17.33 | 16.25 | 15.57 |
| Three or four types | 2.64 | 3.05 | 6.72 | 6.81 |
| Coral reef threat (5 km buffer)  No coral reef along nearest coastline | 25.48 | 26.31 | 27.04 | 27.66 |
| Low | … | … | … |  |
| Moderate | … | … | 3.21 | 3.36 |
| High | 12.12 | 12.46 | 16.41 | 16.25 |
| Very high | 62.40 | 61.24 | 53.35 | 52.73 |
| Mother's educational attainment  No education | 31.82 | 32.32 | 5.47 | 5.74 |
| Primary | 41.53 | 42.51 | 41.05 | 41.76 |
| Secondary or higher | 26.66 | 25.17 | 52.48 | 52.50 |
| Household wealth index  Poorest | 23.46 | 24.48 | 33.95 | 36.02 |
| Poorer | 22.11 | 23.08 | 23.74 | 23.20 |
| Middle | 19.98 | 19.34 | 18.93 | 17.37 |
| Richer | 20.76 | 20.73 | 14.42 | 14.03 |
| Richest | 13.69 | 12.37 | 8.95 | 9.37 |
| Population density quintiles  Lowest population density | 20.37 | 19.60 | 19.00 | 18.98 |
| 2nd quintile | 18.35 | 19.34 | 19.60 | 19.12 |
| 3rd quintile | 19.58 | 19.77 | 20.77 | 20.97 |
| 4th quintile | 19.87 | 18.73 | 20.36 | 20.11 |
| Most densely populated | 21.83 | 22.56 | 20.27 | 20.81 |
| Owns a mode of transportation | 21.62 | 21.17 | 45.50 | 44.67 |
| Total | 1,782 | 1,148 | 7,204 | 4,375 |

Notes: Sample restricted to most recently born child in household to avoid unobservable intrahousehold correlations in selected outcomes.

**Table B.** Percent distribution of DHS sample clusters by distance to coastline

| Distance to coastline | Haiti 2005 | Dominican Republic 2007 |
| --- | --- | --- |
| < 5km | 46.69 | 25.12 |
| 5 – 10 km | 14.76 | 11.44 |
| More than 10 km | 38.55 | 63.44 |
| Total | 332 | 1,425 |

# *Source*: DHS

**Table C.** Frequency distribution of Haiti DHS 2005-2006 sample clusters by level of coral reef diversity and risk (5 km buffer)

|  |  |  | Coral reef risk | |  |  |
| --- | --- | --- | --- | --- | --- | --- |
|  | No coral reef | Low | Medium | High | Very high | Total |
| Coral reef diversity  No coral reef | 66 | … | … | 0 | 0 | 66 |
| One type | 7 | … | … | 16 | 165 | 188 |
| Two types | 0 | … | … | 12 | 59 | 71 |
| Three or four types | 0 | … | … | 6 | 1 | 7 |
| Total | 73 | … | … | 34 | 225 | 332 |

*Source*: WRI Reefs at Risk Indices 2010; UNEP-WCMC Millennium Coral Reef Mapping Project 2010.

**Table D.** Frequency distribution of Dominican Republic DHS 2007 sample clusters by level of coral reef diversity and risk

|  | |  | |  | Coral reef risk | |  | |  | |
| --- | --- | --- | --- | --- | --- | --- | --- | --- | --- | --- |
|  | | No coral reef | | Low | Medium High | | Very high | | Total | |
| Coral reef diversity | |  |  |  |  |  |  | |  |  |
| No coral reef | | 377 | … | 0 | 0 | 0 | 377 | |  |  |
| One type | | 4 | … | 19 | 138 | 542 | 703 | |  |  |
| Two types | | 0 | … | 18 | 67 | 154 | 239 | |  |  |
| Three or four types | | 0 | … | 18 | 41 | 47 | 106 | |  |  |
| Total | | 381 | 0 | 55 | 246 | 743 | 1,425 | |  |  |

*Source*: WRI Reefs at Risk Indices 2010; UNEP-WCMC Millennium Coral Reef Mapping Project 2010.

**Table E.** Estimated odds ratios for the logistic regression model of the relationship between coral reef threat and dietary diversity among children age 6-35 months, Haiti DHS 2005-2006

| Variables Model 1 | | Model 2 | Model 3 | Model 4 | Model 5 | Model 6 |
| --- | --- | --- | --- | --- | --- | --- |
| **Distance to coast line (ref.: < 5 km)**  5-10 km 0.600** | | 0.600** | 0.638* | 0.652* | 0.690 | 0.693 |
| 11-20 km 0.875 | | 0.876 | 0.975 | 0.978 | 0.814 | 0.816 |
| More than 20 km 1.367* | | 1.386 | 1.581** | 1.578** | 1.114 | 1.109 |
| **Coral reef threat (ref.:**  **Very high threat)**  No coral reef |  | 0.989 | 1.017 | 1.006 | 0.989 | 0.984 |
| High threat |  | 1.101 | 1.218 | 1.221 | 0.977 | 0.976 |
| Constant | 0.625*** | 0.620*** | 0.431*** | 0.387*** | 0.611 | 0.614 |
| Wald Chi2 (df) | 10.07 (3) | 10.1 (5) | 15.43 (7) | 20 (11) | 39.1 (15) | 39.7 (16) |
| N | 1,148 | 1,148 | 1,148 | 1,148 | 1,148 | 1,148 |

Notes: Weights included. Models 1-2 have no controls; Model 3 controls for mother’s education; Model 4 controls for household wealth; Model 5 controls for population density; and Model 6 controls for mode of transportation. *** p<0.01, ** p<0.05, * p<0.1.

**Table F.** Estimated odds ratios for the logistic regression model of the relationship between coral reef threat and dietary diversity among children age 6-35 months, Dominican Republic DHS 2007

| Variables Model 1 | | Model 2 | Model 3 | Model 4 | Model 5 | Model 6 |
| --- | --- | --- | --- | --- | --- | --- |
| **Distance to coast line (ref.: < 5 km)**  5-10 km 1.978*** | | 2.021*** | 2.012*** | 2.037*** | 2.114*** | 2.117*** |
| 11-20 km 0.879 | | 0.874 | 0.925 | 0.973 | 1.068 | 1.075 |
| More than 20 km 1.118 | | 1.092 | 1.141 | 1.174 | 1.320** | 1.324** |
| **Coral reef threat**  **(ref.: Very high threat)**  No coral reef | **h** | 0.878 | 0.888 | 0.872 | 0.841 | 0.841 |
| Medium threat |  | 0.902 | 0.894 | 0.889 | 0.929 | 0.920 |
| High threat |  | 0.996 | 1.015 | 1.038 | 1.068 | 1.059 |
| Constant | 2.786*** | 2.920*** | 1.500 | 1.468 | 1.358 | 1.365 |
| Wald Chi2 (df) | 17.11 (3) | 17.9 (6) | 29.5 (8) | 46.0 (12) | 57.7 (16) | 56.2 (17) |
| N | 4,375 | 4,375 | 4,375 | 4,375 | 4,372 | 4,353 |

Notes: Weights included. Models 1-2 have no controls; Model 3 controls for mother’s education; Model 4 controls for household wealth; Model 5 controls for population density; and Model 6 controls for mode of transportation. *** p<0.01, ** p<0.05, * p<0.1.

**Table G.** Estimated odds ratios for the logistic regression model of the relationship between coral reef threat and severe stunting among children age less than 5 years, Haiti DHS 2005-2006

| Variables Model 1 | | Model 2 | Model 3 | Model 4 | Model 5 | Model 6 |
| --- | --- | --- | --- | --- | --- | --- |
| **Distance to coast line (ref.: < 5 km)**  5-10 km 1.804 | | 1.814 | 1.684 | 1.641 | 1.870* | 1.852 |
| 11-20 km 1.905** | | 1.948** | 1.607 | 1.297 | 1.291 | 1.292 |
| More than 20 km 2.017** | | 2.256** | 1.762* | 1.415 | 1.234 | 1.247 |
| **Coral reef threat**  **(ref.: Very high threat)**  No coral reef |  | 0.883 | 0.845 | 0.790 | 0.725 | 0.73 |
| High threat |  | 1.734* | 1.412 | 1.097 | 0.892 | 0.898 |
| Constant | 0.0404*** | 0.0379*** | 0.0653*** | 0.106*** | 0.190*** | 0.188*** |
| Wald Chi2 (df) | 6.753 (3) | 11.49 (5) | 17.69 (7) | 23.23  (11) | 35.55  (15) | 40.83  (16) |
| N | 1,782 | 1,782 | 1,782 | 1,782 | 1,782 | 1,781 |

Notes: Weights included. Models 1-2 have no controls; Model 3 controls for mother’s education; Model 4 controls for household wealth; Model 5 controls for population density; and Model 6 controls for mode of transportation. *** p<0.01, ** p<0.05, * p<0.1.

**Table H.** Estimated odds ratios for the logistic regression model of the relationship between coral reef threat and severe stunting among children age less than 5 years, Dominican Republic DHS 2007

| Variables Model 1 | | Model 2 | Model 3 | Model 4 | Model 5 | Model 6 |
| --- | --- | --- | --- | --- | --- | --- |
| **Distance to coast line (ref.: < 5 km)**  5-10 km 0.375* | | 0.373* | 0.378* | 0.388* | 0.399 | 0.355* |
| 11-20 km 0.912 | | 0.915 | 0.860 | 0.717 | 0.817 | 0.801 |
| More than 20 km 0.827 | | 0.819 | 0.788 | 0.695 | 0.807 | 0.784 |
| **Coral reef threat**  **(ref.: Very high threat)**  No coral reef |  | 1.037 | 1.012 | 0.978 | 0.966 | 0.999 |
| Medium threat |  | 0.614 | 0.630 | 0.601 | 0.610 | 0.577 |
| High threat |  | 1.319 | 1.285 | 1.165 | 1.269 | 1.157 |
| Constant | 0.0179*** | 0.0173*** | 0.0254*** | 0.0341*** | 0.0291*** | 0.0271*** |
| Wald Chi2(df) | 3.006 (3) | 4.876 (6) | 14.86 (8) | 28.42 (12) | 33.08 (16) | 37.66 (17) |
| N | 7,204 | 7,204 | 7,204 | 7,204 | 7,199 | 7,171 |

Notes: Weights included. Models 1-2 have no controls; Model 3 controls for mother’s education; Model 4 controls for household wealth; Model 5 controls for population density; and Model 6 controls for mode of transportation. *** p<0.01, ** p<0.05, * p<0.1.

**Table I.** Estimated odds ratios for the logistic regression model of the relationship between coral reef diversity and dietary diversity among children age 6-35 months living within 5 km of the coastline, Haiti DHS 2005-2006.

| **Variables** | **Model 1** | **Model 2** | **Model 3** | **Model 4** | **Model 5** |
| --- | --- | --- | --- | --- | --- |
| **Index of habitat diversity**  **(ref.: one type of reef)** |  |  |  |  |  |
| No coral reef | 0.762 | 0.811 | 0.768 | 0.755 | 0.735 |
| Two types of reef | 0.505** | 0.507** | 0.553** | 0.490** | 0.493** |
| Three or more types of reef | 0.692 | 0.862 | 0.923 | 0.708 | 0.692 |
| **Constant** | 0.819 | 0.539** | 0.338*** | 0.468 | 0.488 |
| **Wald Chi2 (df)** | 6.766 (3) | 12.51 (5) | 19.75 (9) | 28.76 (13) | 30.48 (14) |
| **N** | 490 | 490 | 490 | 490 | 490 |

Notes: Weights included. Models 1-2 have no controls; Model 3 controls for mother’s education; Model 4 controls for household wealth; Model 5 controls for population density; and Model 6 controls for mode of transportation. *** p<0.01, ** p<0.05, * p<0.1.

**Table J.** Estimated odds ratios for the logistic regression model of the relationship between coral reef diversity and dietary diversity among children age 6-35 months living within 5 km of the coastline, Dominican Republic DHS 2007.

| **Variables** | **Model 1** | **Model 2** | **Model 3** | **Model 4** | **Model 5** |
| --- | --- | --- | --- | --- | --- |
| **Index of habitat diversity**  **(ref.: one type of reef)** |  |  |  |  |  |
| No coral reef | 0.707 | 0.714 | 0.668* | 0.651* | 0.655* |
| Two types of reef | 0.574** | 0.577** | 0.617* | 0.612 | 0.608 |
| Three or more types of reef | 0.537 | 0.548 | 0.611 | 0.520 | 0.523 |
| **Constant** | 3.302*** | 1.770 | 1.888 | 1.615 | 1.581 |
| **Wald Chi2 (df)** | 6.081 (3) | 6.776 (5) | 18.78 (9) | 23.47 (13) | 23.02 (14) |
| **N** | 1,044 | 1,044 | 1,044 | 1,041 | 1,036 |

Notes: Weights included. Models 1-2 have no controls; Model 3 controls for mother’s education; Model 4 controls for household wealth; Model 5 controls for population density; and Model 6 controls for mode of transportation. *** p<0.01, ** p<0.05, * p<0.1.

**Table K.** Estimated odds ratios for the logistic regression model of the relationship between coral reef diversity and severe stunting among children age less than 5 years living within 5 km of the coastline, Haiti DHS 2005-2006.

| **Variables** | **Model 1** | **Model 2** | **Model 3** | **Model 4** | **Model 5** |
| --- | --- | --- | --- | --- | --- |
| **Index of habitat diversity (ref.: one type of reef)** |  |  |  |  |  |
| No coral reef | 1.096 | 1.054 | 1.021 | 0.917 | 0.942 |
| Two types of reef | 1.506 | 1.569 | 1.606 | 1.570 | 1.493 |
| Three or more types of reef | 6.528*** | 5.887*** | 5.686*** | 5.047* | 5.262** |
| Constant | 0.0297*** | 0.0350*** | 0.0353*** | 0.0448*** | 0.0450*** |
| Wald Chi2 (df) | 10.12 (3) | 10.49 (5) | 12.56 (9) | 14.25 (13) | 19.71 (14) |
| N | 760 | 760 | 760 | 760 | 760 |

Notes: Weights included. Models 1-2 have no controls; Model 3 controls for mother’s education; Model 4 controls for household wealth; Model 5 controls for population density; and Model 6 controls for mode of transportation. *** p<0.01, ** p<0.05, * p<0.1.

**Table L.** Estimated odds ratios for the logistic regression model of the relationship between coral reef diversity and severe stunting among children age less than 5 years living within 5 km of the coastline, Dominican Republic DHS 2007.

| **Variables** | **Model 1** | **Model 2** | **Model 3** | **Model 4** | **Model 5** |
| --- | --- | --- | --- | --- | --- |
| **Index of habitat diversity (ref.: one type of reef)** |  |  |  |  |  |
| No coral reef | 0.930 | 0.938 | 1.045 | 1.053 | 1.137 |
| Two types of reef | 1.026 | 1.036 | 0.777 | 0.711 | 0.607 |
| Three or more types of reef | 0.675 | 0.790 | 0.700 | 0.886 | 0.808 |
| **Constant** | 0.0185*** | 0.0351*** | 0.0226*** | 0.0233*** | 0.0180*** |
| **Wald Chi2(df)** | 0.151 (3) | 4.388 (5) | 10.8 (9) | 12.84 (13) | 22.8 (14) |
| **N** | 1,710 | 1,710 | 1,710 | 1,705 | 1,697 |

Notes: Weights included. Models 1-2 have no controls; Model 3 controls for mother’s education; Model 4 controls for household wealth; Model 5 controls for population density; and Model 6 controls for mode of transportation. *** p<0.01, ** p<0.05, * p<0.1.

**Table M.** Estimated odds ratios for the logistic regression model of the relationship between coral reef threat and dietary diversity among children age 6-35 months living within 5 km of the coastline, Haiti DHS 2005-2006

| **Variables** | **Model 1** | **Model 2** | **Model 3** | **Model 4** | **Model 5** |
| --- | --- | --- | --- | --- | --- |
| **Coral reef threat (ref.: Very high threat)** |  |  |  |  |  |
| No coral reef | 1.082 | 1.185 | 1.091 | 0.973 | 0.934 |
| High threat | 1.244 | 1.591 | 1.691 | 0.813 | 0.789 |
| **Constant** | 0.600*** | 0.341*** | 0.184*** | 0.332* | 0.354* |
| **Wald Chi2 (df)** | 0.525 (2) | 6.535 (4) | 15.78 (8) | 23.48 (12) | 26.01 (13) |
| **N** | 490 | 490 | 490 | 490 | 490 |

Notes: Weights included. Models 1-2 have no controls; Model 3 controls for mother’s education; Model 4 controls for household wealth; Model 5 controls for population density; and Model 6 controls for mode of transportation. *** p<0.01, ** p<0.05, * p<0.1.

**Table N.** Estimated odds ratios for the logistic regression model of the relationship between coral reef threat and dietary diversity among children age 6-35 months living within 5 km of the coastline, Dominican Republic DHS 2007

| **Variables** | **Model 1** | **Model 2** | **Model 3** | **Model 4** | **Model 5** |
| --- | --- | --- | --- | --- | --- |
| **Coral reef threat (ref.: Very high threat)** |  |  |  |  |  |
| No coral reef | 0.757 | 0.769 | 0.719 | 0.681* | 0.684 |
| Medium threat | 0.291 | 0.297 | 0.289 | 0.385 | 0.392 |
| High threat | 1.065 | 1.113 | 1.234 | 1.385 | 1.393 |
| **Constant** | 3.081*** | 1.693 | 1.784 | 1.357 | 1.329 |
| **Wald Chi2 (df)** | 3.89 (3) | 4.992 (5) | 17.46 (9) | 22.7 (13) | 22.36 (14) |
| **N** | 1,044 | 1,044 | 1,044 | 1,041 | 1,036 |

Notes: Weights included. Models 1-2 have no controls; Model 3 controls for mother’s education; Model 4 controls for household wealth; Model 5 controls for population density; and Model 6 controls for mode of transportation. *** p<0.01, ** p<0.05, * p<0.1.

**Table O.** Estimated odds ratios for the logistic regression model of the relationship between coral reef threat and severe stunting among children age less than 5 years living within 5km of the coastline, Haiti DHS 2005-2006

| **Variables** | **Model 1** | **Model 2** | **Model 3** | **Model 4** | **Model 5** |
| --- | --- | --- | --- | --- | --- |
| **Coral reef threat**  **(ref.: Very high threat)** |  |  |  |  |  |
| No coral reef | 0.741 | 0.674 | 0.638 | 0.539 | 0.568 |
| High threat | 1.833 | 1.546 | 1.374 | 0.653 | 0.668 |
| **Constant** | 0.0383*** | 0.0509*** | 0.0622*** | 0.196** | 0.188** |
| **Wald Chi2(df)** | 2.172 (2) | 3.074 (4) | 4.892 (8) | 13.99 (12) | 18.86 (13) |
| **N** |  |  |  |  |  |

Notes: Weights included. Models 1-2 have no controls; Model 3 controls for mother’s education; Model 4 controls for household wealth; Model 5 controls for population density; and Model 6 controls for mode of transportation. *** p<0.01, ** p<0.05, * p<0.1.

**Table P.** Estimated odds ratios for the logistic regression model of the relationship between coral reef threat and severe stunting among children age less than 5 years living within 5 km of the coastline, Dominican Republic DHS 2007

| Variables | Model 1 | Model 2 | Model 3 | Model 4 | Model 5 |
| --- | --- | --- | --- | --- | --- |
| **Coral reef threat**  **(ref.: Very high threat)** |  |  |  |  |  |
| No coral reef | 0.950 | 0.970 | 1.095 | 1.091 | 1.200 |
| Medium threat | … | … | … | … | … |
| High threat | 1.177 | 1.365 | 1.167 | 1.346 | 1.470 |
| **Constant** | 0.0181*** | 0.0342*** | 0.0214*** | 0.0197*** | 0.0135*** |
| **Wald Chi2(df)** | 0.101 (2) | 6.212 (4) | 12.93 (8) | 14.21 (12) | 23.5 (13) |
| **N** | 1,700 | 1,700 | 1,700 | 1,695 | 1,687 |

Notes: Weights included. Models 1-2 have no controls; Model 3 controls for mother’s education; Model 4 controls for household wealth; Model 5 controls for population density; and Model 6 controls for mode of transportation. *** p<0.01, ** p<0.05, * p<0.1.
